# Ten simple rules for developing a training program

**DOI:** 10.1371/journal.pcbi.1013200

**Published:** 2025-07-11

**Authors:** Leanna B. Blevins, Amy M. Harrigan, Kevin A. Janes, Jason A. Papin

**Affiliations:** 1 Integrated Translational Health Research Institute of Virginia, University of Virginia, Charlottesville, Virginia, United States of America; 2 Office of the Vice President for Health Sciences and Technology, Virginia Tech, Blacksburg, Virginia, United States of America; 3 Office of the Dean of School of Medicine, University of Virginia, Charlottesville, Virginia, United States of America; 4 Department of Biomedical Engineering, University of Virginia, Charlottesville, Virginia, United States of America; 5 Department of Biochemistry & Molecular Genetics, University of Virginia, Charlottesville, Virginia, United States of America; Carnegie Mellon University, UNITED STATES OF AMERICA

In every facet of a workforce, there is a continual need to develop training programs to update skills and knowledge. Particularly in biomedical and health sciences research, with rapidly changing science comes the need for rapidly developed requisite training. Training programs may be proactive based on institutional aspirations or reactive to institutional needs and/or funder requirements. Target trainees may be pre-professional, early in their careers, or individuals long immersed in a discipline. From our experience leading different training programs, there are several governing principles for the development and implementation of successful training experiences. There are several related Ten Simple Rules articles, including on how to self-train for a new field [1], run workshops [2], and provide bioinformatics training [3,4]. This Ten Simple Rules article represents lessons we’ve learned through success and failure in the development of new training programs. We hope they can be a guide for those wishing to embark on the rewarding journey of training students and professionals to hone their skills and synthesize new information in rapidly evolving fields. Our focus for the rules and examples below is on longer-term (e.g., 1- to 2-year) professional development and training programs for graduate degree students, postdoctoral fellows, and early career faculty. These rules draw on our experience developing a 1-year enrichment program in systems biology and biomolecular data science for PhD students and a 2-year program in clinical translational science for early career faculty. While grounded in these experiences, we emphasize that many of the rules are applicable to a wide range of training programs.

Recruitment–retention of faculty, staff, and a competent workforce are among the biggest investments in research-focused organizations. Professional training often includes the development of leadership capabilities, grant and fellowship writing skills, and statistics and data analysis competency [5], and the continued commitment to education and training in emerging areas makes for more satisfied employees who are more likely to stay [6]. Providing education and professional development to newer or early-career employees is a retention strategy that reinforces the greater needs of the organization.

Training programs can embed within degree-granting graduate programs (e.g., seeking a data science certificate while earning a Ph.D. or M.D.) or as part of an existing role for those already in the workforce (e.g., a mentored career development program for early career researchers). Strong training programs serve as a cornerstone of professional development for current faculty or employees and should be trainee-centered. With our experience in graduate-level and professional training programs, we offer the following guidance to those who are developing their own program.

**1. Pair needs of the target audience with clear training objectives**

Any training program should be aware of the learner population and what skills and knowledge they need for growth and success in their field of study, workforce, or career path. Identify all essential stakeholders, such as funders (e.g., federal, foundation, institutional), institutional leadership, and targeted trainees, and consider their expectations when developing training objectives to increase buy-in and program support. Solidify, document, and communicate the intended trainee outcomes. Defining intended outcomes upfront will enable effective training development and evaluation as well as ensure recruitment of trainees with goals aligned with the program objectives. A response to an overarching call for training programs should be customized to the strengths of an institution. For example, the NIH definition of “biomedical data science” encompasses a large set of activities, but an institution’s training program may appropriately focus on a subset of these activities, which are tailored to the strengths of that institution.

**2. Specify characteristics of potential trainees**

Create trainee inclusion/exclusion criteria that align with the program’s target audience and established training objectives. It is important to lay out these characteristics, the purpose of the training, and all program expectations of trainees when advertising and recruiting for your program. After determining the appropriate trainee background, devise your plan for recruitment, including outreach to diverse participants. Ensure optimal awareness for potential trainees by sharing information to targeted groups/individuals in your professional network and broadly via email distribution lists, newsletters, social media, websites, flyers, etc. Once started, current and past participants are the strongest indicators of a training program—advertise their backgrounds and success outcomes to get more training applicants like them.

**3. Establish a shared leadership structure**

A training program requires a team in which responsibilities are shared among several people. Ideally, a team is composed of complementary subject matter experts relevant to the training objectives. For example, if one of the goals of your training program is to train early career faculty to develop grant writing skills, your team should have one or more members with vast experience writing successful grant applications. It is important to comprise your team of members that can offer varying perspectives while also being thoughtful of the role they will serve to ensure collective input without overlap in responsibilities. To do so, determine the various program aspects when choosing team members, such as financial management, personnel management, curriculum development, trainee management, program evaluation, and resource development. These areas will need leads for guidance and final decisions. Identify the level of administrative support needed for the program and fill that role with qualified personnel. Once roles have been created and filled, ensure everyone understands all roles and responsibilities for the program. These roles and responsibilities should be clearly documented, discussed as a team, and revisited and revaluated when necessary [7]. Our experience suggests that training program leadership with five or fewer individuals ensures effective decision-making processes. Decisions should be made by unanimity as possible; when disagreement arises, it is understood by the leadership team that the program director will make the final decision.

**4. Arrange for instructors that are aligned with the program**

When developing the program curriculum, leverage the expertise within your program leadership team and the organization(s). Recognize that additional external perspectives may need to be incorporated. Trainees will benefit from a diverse set of instructors across different research and academic foci. This inclusion of multiple instructors promotes networking and collaboration across fields which, in turn, expands potential reach. Advisors of the trainees also benefit from the program and thus are incentivized to contribute as instructors or in other roles. Determine incentives your program can offer instructors for their participation. These incentives may be in the form of compensation, if funds are available, a collaborative partnership, or a credit to list in one’s professional portfolio. Consider partnering some curriculum activities with existing programming from other training programs as well to reduce redundancy and resource usage.

**5. Identify and address barriers to participation**

To avoid absenteeism and low engagement, understand the barriers for prospective trainees and devise ways to combat them. For example, time for course and program requirements relative to clinical commitments and existing work responsibilities is a large barrier to training for clinician scientists [5]; expense and travel time are the biggest barriers to Continuing Medical Education (CME) completion [8]. For maximum trainee participation, engage with department chairs, advisors, and other leadership to communicate program expectations before the trainee recruitment and selection phase. Require supervisor support with an application so that trainees are not conflicted when participating in the training amidst other expectations from their supervisors. For example, we require advisors to sign a contract at the onset of training support, which details required program activities and expected time commitment. Investing in relationships with supervisors on the front end of the program alleviates future challenges. Additionally, offering options for alternate participation when conflicts with more than one participant arise will retain higher overall participation levels. Several of our in-person training activities offer a virtual attendance option for trainees who are unable to travel to an event but would like to participate in some way.

**6. Engage learners through effective pedagogy**

A program of professional education for adults should assess what learners already know and what is possible to learn within the experiential component of the program [9]. Trainees benefit from instructors who use proven instructional methods for the content of each session or activity. Additionally, the learning environment (e.g., in-person, virtual, asynchronous) must be considered to develop the most effective training strategy. Because learners often work from multiple dispersed locations, technology is integral to participation and success [10]. For instance, we recognize and evaluate class participation in one of our training courses through impromptu questions and answers and open-ended chats on a live discussion board. Other examples include pre-work, group work, inverted sessions (where the designated course time is used for discussion and content is learned beforehand independently), and mixed media materials. Ensure that training structures are accessible and equitable. Lastly, even after effectively communicating the program expectations during the trainee recruitment and selection phase (Rules #2 and #5), it is important to develop, communicate, and follow a policy that allows for excused absences, as defined by the program (e.g., parental leave, jury duty), and a maximum number of unexcused absences. This policy should be based on the impact absences have on both a trainee’s and the program’s training goals; our experience suggests that trainees with participation in at least 80% of the program offerings are trained sufficiently to meet program objectives and that the trainee cohort maintains a strong sense of community.

**7. Create camaraderie within the training cohort**

Create an inclusive cohort so that no individual feels alone or excluded. Trainees both contribute to and benefit from the entire cohort [11], so optimizing engagement in both learning and the cohort community are crucial. Include activities that build cohesion among the trainees, especially in the early sessions. For instance, an online program may consider starting each program meeting/session with a short icebreaker to encourage engagement and socialization. Additionally, offering a few optional in-person sessions that supplement online training may help strengthen cohort connections. Help trainees develop a trainee identity at the institution and/or on social media and encourage them to interact with one another using those platforms. Consider a group project for trainees that will result in something mutually beneficial to all members (such as a publication). There are challenges establishing collaborations between various groups [12] and thus it is critical to be deliberately mindful of the relationship-building process. Doing so will increase professional productivity. Icons are remarkably helpful for building a sense of identity. We’ve seen former trainees proudly wear sweatshirts, display stickers, and use coffee mugs emblazoned with the logo of our programs. These visuals advertise that identity to future program applicants.

**8. Document and organize everything well**

Accessibility of the program materials is central to success. For example, learners should have the calendar of events and materials for all training sessions, and leadership teams should have records of attendance and access to real-time evaluation data. To establish this practice, use institution-supported, secure cloud servers (as available) that restrict access to users with permissions to store all program files and give access to those that need it. Maintenance of these files is crucial in multiple ways and having qualified administrative support, as mentioned in Rule #3, will make it much more likely to be successful at this rule. Accurate, up-to-date documentation and sensible organization of program artifacts supports engagement, reduces unnecessary complexity, and maximizes attention to activities aligned with the goals of the program. The ability to assess, adjust, and adapt (Rule #9) relies on thorough documentation and systematic organization to facilitate timely identification of program and/or trainee needs that may require immediate attention and/or remediation.

**9. Assess, adjust, adapt**

When establishing training goals and a program curriculum, devise a plan to assess the intended outcomes. There is a rich literature on best practices for assessing education programs in science [13]. Consider multiple forms of evaluations, such as objective data, direct observation from program leadership, and feedback from instructors, trainees, and institutional leaders. Give trainees the opportunity to provide anonymous, regular, and consistent feedback on key elements of the program. We favor a Likert scale [14] ranging from “Strongly Agree” (high point), “Agree”, “Neither Agree nor Disagree” (neutral middle point), ”Disagree”, and “Strongly Disagree” (low point). This type of scale quantifies feedback for aggregation and analysis of historical trends. Short surveys implemented at every training session provides specific feedback on the content that is considered in future offerings. More detailed surveys implemented every 6–12 months provide valuable feedback for higher-level program design. Participation in surveys should be required by the trainees and communicating how the feedback is used will provide motivation for frank and constructive responses. Make a plan for program leadership to formally and informally check in with trainees individually at regular intervals. Assessment data should be discussed with program leadership to identify needed modifications. Programmatically, do not be afraid to cut out the aspects that simply are not effective. Avoid the trap of continuing something solely because it has been done before.

**10. Foster sustained support of the program, but also know when to quit**

Trainees need support incorporating what they learn into their work [5]. Provide resources and guidance to trainees on how to optimize the knowledge and skills learned during training. Ensure supervisors of trainees are aware of program content and objectives so that they provide support external to the program and trainees are better able to integrate lessons learned into their work. As their formal engagement with the program ends, track outcomes of the trainees; pair this data with program assessment data to help justify continued and new support from funders and institutional leadership. Consider how the trainees will engage with the program after their formal training has ended; they will be your greatest advocates. Funder and institutional priorities or needs change over time. Take time to step back and determine if the program is still needed or wanted and by whom. Clearly establishing goals from the beginning (Rule #1) is essential for assessing impact of the program (Rule #9); alignment or misalignment of training goals with program assessments will inform decisions about the continuation or sunsetting of the program.

In the ever-changing landscape of scientific discovery, those who prioritize a culture of learning and development are better positioned to navigate challenges and seize opportunities to innovate. Investing in the growth of trainees and faculty toward their implementation of new knowledge and skills not only benefits the individuals but also contributes to the long-term success and sustainability of the scientific community as a whole. Enabling the successful start-up of a training program by utilizing demonstrated best practices allows new programs to accelerate the learning curve and focus on trainee development and achievements, which is the goal of all educators.
